# An Atypical Case of Spondylitis Due to Nontyphoidal Salmonella in an Adult Patient: A Case Report and Review of the Literature

**DOI:** 10.7759/cureus.55034

**Published:** 2024-02-27

**Authors:** Toru Hamada, Shinya Furukawa, Yoshio Kikuchi, Masashi Kubota, Eiji Mitsunaga

**Affiliations:** 1 Internal Medicine, Seiyo Municipal Hospital, Seiyo, JPN; 2 Health Services Center, Ehime University, Matsuyama, JPN; 3 Orthopedics, Seiyo Municipal Hospital, Seiyo, JPN

**Keywords:** localized pain, non-steroidal anti-inflammatory, blood cultures, spondylitis, non-typhoidal salmonella

## Abstract

Nontyphoidal Salmonella commonly induces intestinal infections; however, spondylitis arising from this bacterium is exceedingly rare. A comprehensive review of the clinical attributes of nontyphoidal Salmonella-induced spondylitis in adult populations is lacking in the literature. We report a case of an 83-year-old female who presented with a fever lasting three days, accompanied by anorexia and pervasive malaise. A month prior, she had been prescribed celecoxib and had received a trigger point injection. The patient was initially diagnosed with acute pyelonephritis and treated with an antimicrobial regimen. However, a week later, although her fever persisted, there was no complaint of back pain. The discontinuation of celecoxib led to back pain. Subsequent urine and blood cultures, coupled with MRI findings, confirmed the diagnosis of pyogenic spondylitis attributable to the Salmonella O7 group. The patient’s fever abated with the administration of antimicrobial agents, and her back pain subsided. The antimicrobial regimen was continued for 12 weeks, with no resurgence of fever or back pain following treatment.

Local pain and fever are important indicators for the diagnosis of spondylitis caused by nontyphoidal Salmonella. It is critical to take an accurate history of non-steroidal anti-inflammatory drugs (NSAIDs) use, such as celecoxib, since NSAIDs can obscure the symptoms. Blood cultures are equally important, given their propensity to yield positive results in these patients.

## Introduction

The genus Salmonella encompasses a diverse array of serotypes, and those distinct from Salmonella Typhi and Salmonella Paratyphi A fall within the category of nontyphoidal Salmonella. Nontyphoidal Salmonella primarily causes enteric infections, although spondylitis, an infrequent complication, has also been documented [1]. A comprehensive review of the clinical features of spondylitis induced by nontyphoidal Salmonella in children has been published [2] and multiple case reports have documented spondylitis induced by nontyphoidal Salmonella in adults; however, no comprehensive review encompassing the clinical features exists in the literature currently. Historically, localized pain (in the chest, back, or lumbar region) is observed in most cases of spondylitis caused by nontyphoidal Salmonella. Spondylitis is typically diagnosed when patients present with a complaint of localized pain, prompting blood culture and disc culture assessments to identify the causative organism, alongside MRI and other imaging studies to corroborate the consistent indications of spondylitis. We discuss a case of a patient with spondylitis attributed to nontyphoidal Salmonella, which was initially unrecognized as the patient did not complain of localized pain (potentially attributed to analgesic use), which led to a diagnostic delay.

This article was previously posted to the Research Square preprint server on January 8, 2024.

## Case presentation

An 83-year-old female presented to our hospital with persistent fever for three days, along with anorexia and general malaise. She reported maintaining an independent lifestyle and was actively engaged in daily fieldwork. Her medical history included hypertension, dyslipidemia, reflux esophagitis, and insomnia. She was taking diltiazem, imidafenadine (a urinary antispasmodic), isosorbide, pravastatin, lansoprazole, clotiazepam, and celecoxib. She had no history of allergies, alcohol consumption, or smoking. Approximately one month before her hospitalization, she had experienced a heat stroke concurrent with the onset of back pain, for which she had sought treatment from a local orthopedic surgeon and had been prescribed celecoxib. She had also received regular trigger point injections in her lumbar back area.

Upon initial presentation at our hospital, the patient had a fever of 38 °C without back pain, lumbago, dysuria, or costovertebral angle tenderness. She had no complaints of abdominal pain, diarrhea, or hematochezia. The patient did not manifest conjunctival hyperemia, ocular conjunctival yellowing, generalized joint pain, or extremity numbness. Laboratory investigations revealed a white blood cell (WBC) count of 8860/µL and a C-reactive protein (CRP) level of 8.57 mg/dL. Although she had not been previously diagnosed with diabetes mellitus, blood tests indicated elevated glucose levels (136 mg/dL) and an HbA1c level of 7.4% (reference range: 4.6-6.2%). Moreover, her urine exhibited bacterial contamination. A CT scan revealed no signs of hydronephrosis or inflammatory etiology. Following the submission of urine and blood cultures, she was treated as an outpatient for acute pyelonephritis and prescribed amoxicillin-clavulanic acid (AMPC-CVA). Despite commencing AMPC-CVA, her fever and fatigue persisted. Five days after her initial hospital visit, she presented again with improved appetite but with persistent malaise and a sustained fever of 38 °C. Notably, the patient did not report back pain, backache, or joint pain. She had no gastrointestinal symptoms, such as diarrhea, abdominal pain, lower limb numbness, or voiding issues. Subsequent blood tests showed a declining inflammatory response with a WBC count of 7710/μL and a CRP of 5.52 mg/dL. She was admitted due to continued malaise and inability to be managed at home.

**Figure 1:**
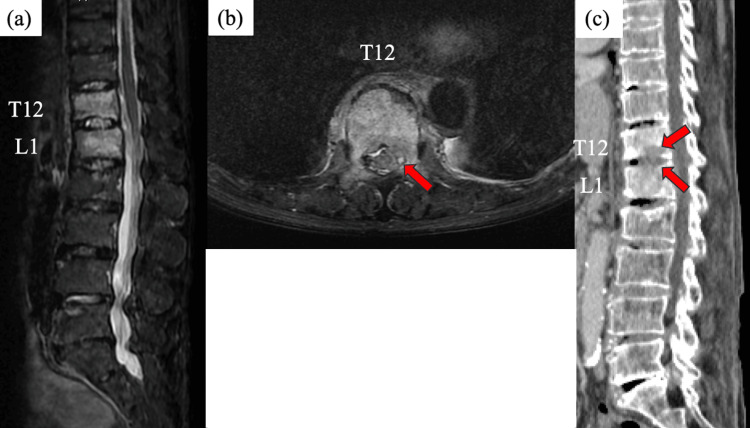
Image examination (a) The sagittal section of the spinal MRI on day one revealed high-signal alterations consistent with pyogenic spondylitis in the T12 and L1 vertebrae. (b) The axial section of the spinal MRI at the T12 level on day X+1 revealed a high-signal region within the spinal canal, suggestive of an epidural abscess. (c) The sagittal section of the CT scan on day X-5 revealed osteolytic alterations in the T12 and L1 vertebral bodies CT: computed tomography; MRI: magnetic resonance imaging

On admission, celecoxib was discontinued to evaluate the patient’s fever pattern; urine and blood cultures had confirmed the presence of Salmonella O7. Contrast-enhanced CT showed no evidence of abscess or infected aneurysm. Transthoracic echocardiography showed no findings indicative of verrucae. On day one, the radiologic evaluation revealed osteolytic changes in the spine (T12 and L1 level) on the initial CT scan. A subsequent spine MRI showed high-signal areas in the vertebral bodies at the T12 and L1 levels and the intervertebral discs at the T12/L1 level (Figure 1).

**Figure 2:**
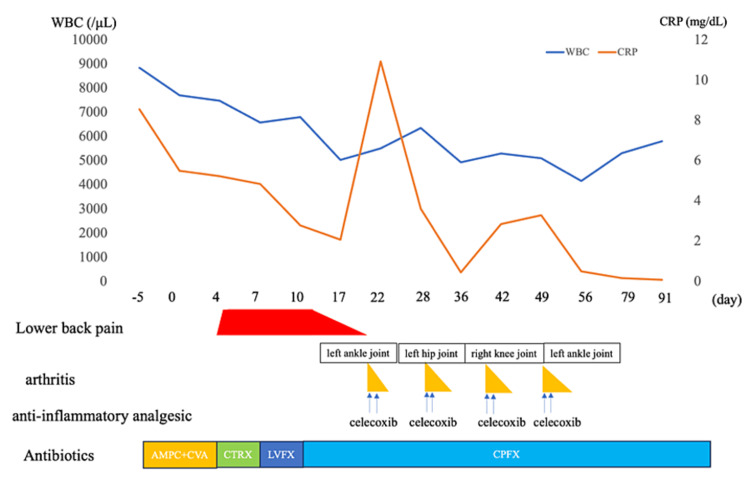
Clinical course following treatment initiation WBC: white blood cell; CRP: C-reactive protein; AMPC: amoxicillin 500 mg q8h; CVA: clavulanic acid 125 mg q8h; CTRX: ceftriaxone 2 g q24h; LVFX: levofloxacin 500 mg q24h; CPFX: ciprofloxacin 400 mg q12h

A diagnosis of pyogenic spondylitis due to Salmonella O7 was made. Salmonella is susceptible to AMPC-CVA, ceftriaxone (CTRX), ciprofloxacin (CPFX), and levofloxacin (LVFX). Conservative treatment with CTRX was initiated because of the absence of nerve compression symptoms. On day three, lower back pain was observed during body movement, but subsequent blood cultures on day four were negative; on day eight, treatment was changed from LVFX to CPFX; on day nine, the patient became fever-free; on day 15, her back pain disappeared. However, on day 21, she again developed a fever of 39 °C, and her left ankle joint showed inflammatory findings, including fever and redness, but she reported no worsening of back pain. On day 22, she was treated with celecoxib. On day 26, the arthritis in the left ankle resolved. Subsequently, arthritis developed successively in the left hip, right knee, and left ankle joints, all of which were treated with celecoxib. Although celecoxib was used in this case to treat recurrent crystalline arthritis, it was inconclusive whether this arthritis could be distinguished from reactive arthritis. Despite the multiple recurrences of arthritis, the patient did not have recurrent back pain, suggesting that the spondylitis was adequately managed. The patient was discharged on day 58 and reported free of fever and arthralgia on day 79. CPFX therapy was continued for 12 weeks (until day X+91) with the resolution of fever and back pain. After antimicrobial therapy, the patient’s WBC counts and CRP levels were found to be normal. At the three-week follow-up (day 113) after CPFX was discontinued, the patient reported no fever or pain (Figure 2).

## Discussion

Salmonella is a gram-negative, facultative anaerobic bacterium with serotypes other than Salmonella Typhi and Salmonella Paratyphi A; the other types are categorized as nontyphoidal Salmonella. Typhoidal Salmonella infections tend to provoke severe gastrointestinal symptoms, whereas nontyphoidal Salmonella infections typically result in mild symptoms. Both types can lead to systemic infections that rarely escalate to osteomyelitis [3]. Of these, an even smaller fraction culminates in spondylitis [2]. Several studies have explored spondylitis caused by typhoidal Salmonella and described its clinical characteristics [4]. Correspondingly, there have been reviews examining spondylitis caused by nontyphoidal Salmonella spp. in children [2], delineating typical clinical presentations. However, although there are 21 case reports of spondylitis due to nontyphoidal Salmonella in adults published in English on PubMed, comprehensive reviews of its clinical features are lacking [5-25].

Our case differs from previous cases of spondylitis caused by nontyphoidal Salmonella in two ways. Firstly, in the present case, local pain, commonly associated with nontyphoidal Salmonella spondylitis, was subdued by oral non-steroidal anti-inflammatory drugs (NSAIDs) and trigger point injections. Nontyphoidal Salmonella spondylitis is rare in adults. Initially, we reviewed all cases of Salmonella-associated spondylitis reported in English with full texts available on PubMed and the Central Journal of Medicine, limited to adults 19 years and above. This search yielded 30 cases [5-34], 21 of which were identified as nontyphoidal Salmonella [5-25]. Of these, 17/21 (81.0%) patients reported local pain, and only 4/21 (19.0%) had no local pain (one case presented with impaired consciousness, one with spinal cord/peripheral nerve palsy, one with no clear identified cause, and one had no description provided). In the present case, the use of NSAIDs masked the spondylitis pain during the initial visit, and back pain emerged once NSAIDs were discontinued after admission. NSAIDs are often prescribed as antipyretic analgesics, and their use is particularly frequent in the elderly [35]. A comprehensive drug history is especially important in elderly patients, such as in our case, because the pain may be masked by the patient’s use of NSAIDs at the time of the initial visit.

Secondly, this is the first case report describing the clinical manifestations of spondylitis caused by nontyphoidal Salmonella in adults. The clinical characteristics of 22 spondylitis cases caused by nontyphoidal Salmonella, including the present case, are outlined in Table 1. The average age of these patients was 53.4 (20-85) years, and 6/22 patients (27.3%) were female. A history of consuming raw food and gastrointestinal symptoms, such as diarrhea and abdominal pain, were evident in 4/22 cases (18.2%). Moreover, 17/22 patients (77.3%) reported local pain during their initial visit, whereas those without local pain might have been asymptomatic due to impaired consciousness or neurological disorders. Fever was noted in 19/22 cases (86.4%), and blood cultures were positive in 18/22 cases (81.8%), demonstrating their value in diagnosing spondylitis. Additionally, 4/22 cases (18.2%) exhibited culture-positive abscesses or intervertebral discs in surgical specimens. Although stool cultures were positive in 4/22 cases (18.2%), stool cultures were not obtained in several instances. The serotypes of the causative organisms varied and are listed in Table 1.

**Table 1:** A review of case reports of spondylitis due to nontyphoidal Salmonella in adults (including the present case) *0 = none, 1 = yes; **1 = blood, 2 = biopsy, and 3 = stool; ***X = X-ray, C = CT, and M = MRI CT: computed tomography; MRI: magnetic resonance imaging

Age in years/sex	Local pain*	Fever*	Cultures	Cultures found positive**	Lesion (device)***	Pathogenic bacteria	Time to medical exam (days)	Treatment period (weeks)	Surgery or drainage*	Reference
22/M	1	1	Positive	1, 3	No specific mention (M)	Salmonella enterica	2	NA	NA	[5]
54/M	1	1	Positive	1, 3	L3-S2 (M)	Salmonella enterica ser. Enteritidis	5	16	0	[6]
29/F	1	0	Positive	3	L4/5 (X, M)	Salmonella Potsdam	50	6	1	[7]
59/M	0	1	Positive	1	T8/9 (M)	Salmonella enterica subsp. enterica ser. Enteritidis	11	16	0	[8]
65/M	1	0	Positive	1, 2	L5/S1 (M)	Salmonella enterica	NA	8	1	[9]
54/M	0	1	Positive	1	T2/T3, T5/T6 (M)	Salmonella enterica ser. Typhimurium	3	8	0	[10]
50/M	1	1	Positive	1	L2/3 (M)	Salmonella enteritidis O group ser. E4 ser. Westerstede	2	12	0	[11]
52/M	1	1	Positive	1	L4/5 (M)	Salmonella Typhimurium (phage type 104)	180	12	0	[12]
56/M	1	1	Positive	1, 2, 3	L1-4 (M)	Salmonella enterica ser. Cholerae	10	6	1 (drainage of abscesses)	[13]
44/M	0	1	Positive	1, 3	T3-9 (C)	Salmonella Paratyphi B	28	3 or more	0	[14]
60/M	1	1	Positive	1	L4/5 (M)	Salmonella choleraesuis	3	3 or more	0	[15]
75/F	1	1	Positive	1	T10/11, L2/3 (M)	Salmonella Newport	2	18	0	[16]
20/M	1	1	Positive	1	No specific mention (M)	Salmonella group C	7	6	1	[17]
42/M	1	1	Positive	2	T12 (X, C, M)	Salmonella group B	28	6	1	[18]
37/M	1	1	Positive	1	C7-T1 (X)	Salmonella Utah	21	10	0	[19]
33/F	1	1	Positive	1	L3/4 (X)	Salmonella choleraesuis	14	NA	1 (anterior lumbar interbody fusion)	[20]
68/M	1	1	Positive	1, 2	T9/10 (C, M)	Salmonella enteritidis (O9)	24	5	1 (posterior lumbar interbody fusion)	[21]
85/F	1	1	Positive	1	L4/5 (X, M)	Salmonella serogroup C2	1	10	1	[22]
54/F	1	1	NA	NA	L2/3 (C)	Salmonella Corvallis	540	NA	0	[23]
80/M	1	1	Positive	1	L3/4 (M)	Salmonella enteritidis	7	lifetime	1 (endovascular aortic repair)	[24]
53/F	NA	NA	Positive	2	T8-10 (M)	Salmonella Typhimurium	30	6	1 (posterior lumbar interbody fusion)	[25]
83/F	0	1	Positive	1	T12/L1 (C, M)	Salmonella O7	30	12	0	Current case

Infection sites were identified via X-ray, CT, and MRI, with multiple overlapping sites: cervical spine in 1/22 cases (4.5%), thoracic spine in 9/22 cases (40.9%), lumbar spine in 13/22 cases (59.1%), and unidentified sites in 2/22 cases (9.1%). Additionally, 4/22 cases (18.2%) presented with concomitant spinal epidural abscesses. The average duration from symptom onset to consultation was 22.9 days, whereas the duration of antimicrobial treatment varied. Overall, 8/22 cases (36.4%) required drainage of the infected lesions.

The significance of local pain and clinical presentations in nontyphoidal Salmonella-induced spondylitis was emphasized in these cases. However, when NSAIDs have already been administered, as in the present case, the crucial symptom of local pain could be camouflaged, hindering the diagnosis of spondylitis. NSAIDs are frequently prescribed to the elderly because of prevalent back pain [35], with some patients receiving prolonged courses. In our case, a subsequent interview revealed that the patient had experienced lower back pain and started taking NSAIDs a month before visiting our clinic. Based on blood culture results and CT and MRI findings, we arrived at a diagnosis of spondylitis.

## Conclusions

The presence of local pain can be masked in patients taking NSAIDs, necessitating a thorough review of drug history, even if patients do not verbally express the presence of local pain. Furthermore, the presence of fever and blood culture results are useful in diagnosing spondylitis, and the detection of nontyphoidal Salmonella in blood cultures warrants the consideration of spondylitis.
